# Evaluation of balloon pulmonary angioplasty using lung perfusion SPECT in patients with chronic thromboembolic pulmonary hypertension

**DOI:** 10.1007/s12350-022-02971-0

**Published:** 2022-04-26

**Authors:** Hidenobu Hashimoto, Takashi Oka, Rine Nakanishi, Sunao Mizumura, Shintaro Dobashi, Yukiko Hashimoto, Yuriko Okamura, Kyoko Ota, Takanori Ikeda

**Affiliations:** 1 Department of Cardiovascular Medicine, Department of Internal Medicine, Faculty of Medicine, Toho University, 6-11-1, Omorinishi, Ota-ward, Tokyo, 143-8541 Japan; 2 Department of Radiology, Faculty of Medicine, Toho University, Tokyo, Japan

**Keywords:** SPECT, hybrid imaging, CT, vascular imaging, perfusion agents

## Abstract

**Background:**

The aim of this study was to evaluate the effect of balloon pulmonary angioplasty (BPA) using lung perfusion single-photon emission computed tomography (SPECT) in patients with chronic thromboembolic pulmonary hypertension (CTEPH).

**Methods and Results:**

20 consecutive patients (64 ± 15 years) who were diagnosed with CTEPH and underwent BPA were included in this study. All patients underwent lung perfusion SPECT before and after BPA. The relationship between functional %volume of the lung calculated from the lung perfusion SPECT (FVL-LPSPECT), and other clinical parameters before and after BPA was assessed using the Wilcoxon signed-rank test. The correlation between each parameter and mean pulmonary artery pressure (mPAP) using the Spearman’s correlation was performed. To determine predictors of mPAP for evaluating treatment effectiveness, significant parameters were included in multiple regression analysis. After BPA, world health organization functional classification, six-minute walk distance (6MWD), mPAP, and FVL-LPSPECT significantly improved. FVL-LPSPECT (*r* = − 0.728, *P* < 0.001) and 6MWD (*r* = − 0.571, *P* = 0.009) were significant correlation of mPAP. In the multiple regression analysis, FVL-LPSPECT was the most significant predictor of improvement in mPAP after BPA (*P* < 0.001).

**Conclusions:**

This study demonstrated that the lung perfusion SPECT could be a potential measurement of the effectiveness of BPA in patients with CTEPH.

**Supplementary Information:**

The online version contains supplementary material available at 10.1007/s12350-022-02971-0.

## Introduction

The annual number of patients with acute pulmonary embolism in the United States is estimated at 600,000.^1,2^ Chronic thromboembolic pulmonary hypertension (CTEPH) develops in approximately 3.8% of patients surviving the acute phase of pulmonary embolism.^2,3^ The pathology of CTEPH is related to increased flow resistance through the pulmonary circulation that results from obstruction of the pulmonary arteries by thromboembolism and vascular remodeling.^4^ Increased pulmonary arterial pressure (PAP) leads to right ventricular pressure overload and dysfunction and is associated with considerable mortality.^5^ Patients with a severe grade of CTEPH have a poor prognosis; the 5-year-mortality rate is reported to be more than 50%.^6^ More than a third of patients with CTEPH are considered inoperable.^7^

In recent years, the therapeutic options for patients of CTEPH with technically inoperable disease have expanded with balloon pulmonary angioplasty (BPA).^8^ Moreover, there has been a significant improvement in the prognosis of CTEPH.^9^ However, the quantitative measurement of treatment effectiveness in patients with CTEPH using imaging modalities has not been well-established. In the present study, we aimed to investigate the effectiveness of BPA in patients with CTEPH using lung perfusion single-photon emission computed tomography (SPECT).

## Methods

### Patient population

**Figure 1:**
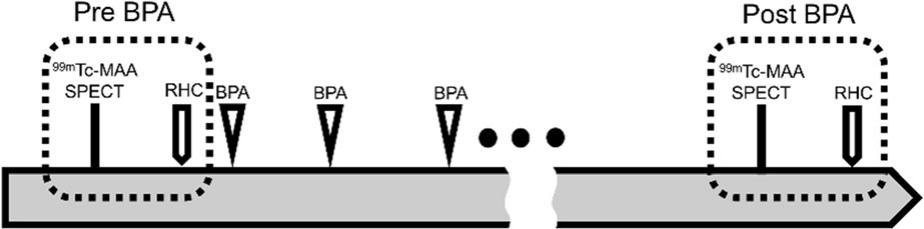
Study outline. ^99m^Tc-MAA SPECT, technetium-99m-macroaggregentated albumin single-photon emission computed tomography; RHC, right heart catheterization; BPA, balloon pulmonary angioplasty

A total of 20 consecutive patients diagnosed with CTEPH between January 2017 and January 2021 were included in this study. They were diagnosed as having inoperable CTEPH based on the standard criteria.^10^ All participants underwent BPA, and lung perfusion SPECT and chest computed tomography (CT) were performed before and after BPA (Figure 1). We assessed the patients’ clinical characteristics, including age, sex, medical history, blood biochemistry data, echocardiography data, and medications. The Ethics Committee of Toho University Omori Medical Center approved this study, and all individuals provided written informed consent (M20307 20075 17187).

### Echocardiographic imaging

Echocardiographic images were obtained from the apical window to evaluate the right ventricular function (Vivid E9device, GE Vingmed, Horten, Norway). Tricuspid regurgitation pressure gradient was calculated.^11^

### Right heart catheterization

All participants underwent right heart catheterization in a supine position before BPA. Experienced interventionalists assessed the quantitative hemodynamics, including mixed venous oxygen saturation, mean pulmonary arterial pressure (mPAP), cardiac index, and pulmonary vascular resistance (PVR).

### Balloon pulmonary angioplasty

BPA was performed via the right femoral vein to treat pulmonary arterial branches. We selected vessels appropriate for ballooning based on comprehensive findings, including the presence of webs, bands, abrupt narrowing, and complete obstructions as seen on pulmonary angiography. Under continuous intravenous infusion of heparin, we used a 0.035-inch wire (Large Focus, Terumo, Tokyo, Japan) advanced to the targeted vessels for appropriate guiding of the catheters (JR or AL; Boston Scientific, Natick, MA, USA). We selected the balloon size based on a targeted vessel diameter measured by angiography. After crossing a 0.014-inch guidewire (B-pahm 0.6, Japan Lifeline Co., Ltd, Tokyo, Japan) through the lesion, we carefully inflated the balloon (Sapphire II PRO PTA for a lesion of 1.0–2.5 mm in diameter, OrbusNeich, Hong Kong, China; Crospander for a lesion of 3.0–4.0 mm in diameter, Japan Lifeline Co., Ltd, Tokyo, Japan; Bandiccot RX for a lesion of 5.0–7.0 mm in diameter, Kaneka Medical Products, Osaka, Japan; Makaira for a lesion of 8.0 mm in diameter, Fukuda Denshi, Tokyo, Japan). Prompt visualization of the pulmonary venous flow through the treated pulmonary arteries was defined as success. The BPA procedures were repeated at an interval of 4–8 weeks, and additional BPA was repeated until mean PAP decreased below 30 mmHg or no more treatable lesions were found.

### Lung perfusion SPECT

Lung perfusion SPECT was performed using a dual-head gamma camera (Infinia, GE Healthcare, Buckinghamshire, UK) equipped with low-energy, high-resolution collimators within 14 days before and 28–56 days after BPA. SPECT data were acquired using 72 projections over an orbit of 360° per step and 15 seconds per projection for five minutes after injecting 370MBq of technetium-99m-macroaggregated albumin (^99m^Tc-MAA) with the patient in the supine position. The image matrix size was 128 × 128. The image reconstruction was performed using filtered back projection and processed with Butterworth prefiltering (critical frequency 0.5, power 10.0).

### Chest CT

Chest CT data performed within 30 days before BPA were used (Aquilion Precision; Canon medical systems, Tokyo, Japan). Scan parameters were as follows: tube voltage, 100 kV at auto mA; rotation time, 0.33 second; collimation, 160 × 0.25 mm; pich, 1.388. All scans were reconstructed as 5.0 mm thick slices with an increment of 5.0 mm.

### Functional volume of lungs calculated from lung perfusion SPECT

**Figure 2:**
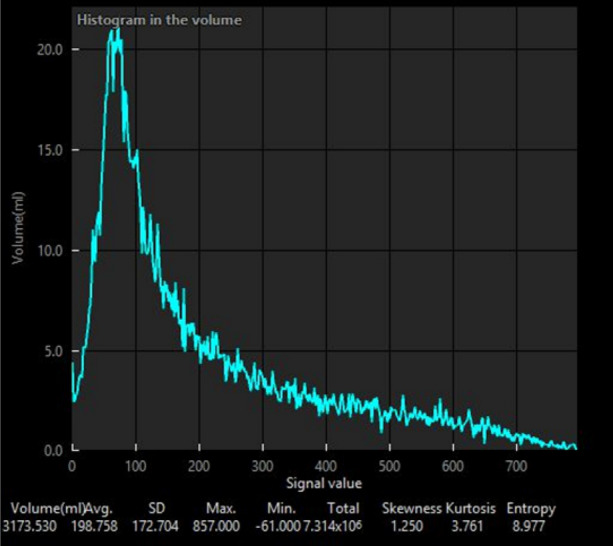
A histogram of radioactivity volume generated from ^99m^Tc-MAA SPECT. ^99m^Tc-MAA SPECT, technetium-99m-macroaggregentated albumin single-photon emission computed tomography

Lung perfusion SPECT images and chest CT images were analyzed using a workstation (SYNAPSE VINCENT; FUJIFILM Medical Co., Ltd, Tokyo, Japan). The functional volume of lungs was defined as the volume on the lung perfusion SPECT; the edge of the lung perfusion SPECT was determined at 10% of the maximum counts using a histogram (Figure 2). The lung volume as the total functional volume of the lung was defined as the volume of the lung seen on chest CT. Both, functional volume of the lung and lung volume were calculated using the workstation. Functional %volume of lung calculated from the lung perfusion SPECT (FVL-LPSPECT), which indicated the preservation rate of normal functional lung area, was calculated by dividing the functional volume of the lungs calculated from the lung perfusion SPECT, by the lung volume calculated from the chest CT.

### Statistical analysis

Data of continuous variables are expressed as average ± standard deviation. Various clinical parameters, including blood test, echocardiography, right heart catheterization, and lung perfusion SPECT findings of patients before and after BPA, were compared using the Wilcoxon signed-rank test. The correlations between each parameter and mPAP, six-minute walk distance (6MWD), and PVR as markers of BPA treatment effectiveness were calculated using Spearman’s correlation. To determine markers of treatment effectiveness, significant parameters were subsequently included in each multiple regression analysis. A *P* value of < 0.05 was considered statistically significant. All statistical analyses were performed using StatMate V software version 5.01 (Advanced Technology for Medicine and Science, Tokyo, Japan).

## Results

The patients’ characteristics, including coronary risk factors, number of BPA sessions, medication for CTEPH, are presented in Table 1. The mean age of the 20 patients was 64 ± 15 years, and six (30%) of them were male. Hypertension was the most common complication (*n* = 4, 20%). The duration of pulmonary embolism from the index episode in patients with CTEPH in this study was 48.7 ± 83.0 months, and that of pulmonary hypertension was 18.3 ± 34.4 months. In this study, all patients were considered inoperable because the lesions of the pulmonary arteries were considered peripheral diseases. The number of BPA sessions was 6.0 ± 2.2.

**Table 1:** Patients characteristics

	Total (n = 20, %)
Age (years)	64 ± 15
Male	6 (30)
Obesity (BMI ≥ 30kg/m^2^)	3 (15)
BMI (kg/m^2^)	23.5 ± 5.6
Diabetes mellitus	3 (15)
Hypertension	4 (20)
Dyslipidemia	3 (15)
Current smoking	6 (30)
CKD	2 (10)
*BPA*	
Number of BPA sessions	6.0 ± 2.2
*Medication*	
Endothelin receptor antagonist	2 (10)
Prostaglandin I_2_	1 (5)
Phosphodiesterase type 5 inhibitor	1 (5)
Stimulators of soluble guanylate cyclase	7 (35)
Direct oral anticoagulant	15 (75)
Warfarin	5 (25)

BMI, body mass index; CKD, chronic kidney disease; eGFR, estimated glomerular filtration rate; BPA, balloon pulmonary angioplasty

During a mean follow-up of 694 ± 328 days, compared to before BPA, there was a significant improvement in clinical parameters including FVL-LPSPECT after BPA (world health organization functional classification (WHO FC): 2.3 vs. 1.3, *P* < 0.001, 6MWD (m): 303.8 vs. 427.2, *P* < 0.001, mPAP (mmHg): 40.2 vs. 23.2, *P* < 0.001, FVL-LPSPECT (%): 62.2 vs. 87.3, *P* < 0.001) (Table 2). No patients exhibited complications after BPA sessions. Additionally, there were no cases that underwent thrombectomy in this study. Regarding Spearman’s correlation, FVL-LPSPECT and 6MWD were identified as markers of improvement in mPAP. FVL-LPSPECT was the most significant marker for mPAP improvement after BPA (*P* < 0.001) (Table 3). mPAP was a significant marker for improvement in 6MWD after BPA (Table 4). There was no significant marker for PVR improvement after BPA (Table 5).

**Table 2:** Clinical parameters and LPSPECT parameters before and after BPA

	Before BPA	After BPA	P value
*Physical examination*			
WHO FC	2.3 ± 0.6	1.3 ± 0.5	<0.001
6MWD (m)	303.8 ± 182.0	427.2 ± 138.3	<0.001
*Blood test*			
BNP (pg/mL)	284.6 ± 387.8	44.2 ± 71.4	0.005
*Echocardiography*			
TRPG (mmHg)	51.8 ± 20.7	24.0 ± 10.4	<0.001
*Right heart catheterization*			
SvO_2_ (%)	57.4 ± 12.3	67.2 ± 5.2	0.005
mPAP (mmHg)	40.2 ± 7.8	23.2 ± 5.1	<0.001
CI (L/min/m^2^)	3.3 ± 0.8	3.1 ± 0.6	0.911
PVR (dyne s cm^−5^)	568.5 ± 207.5	255.5 ± 78.8	<0.001
*LPSPECT*			
FVL-LPSPECT (%)	62.2 ± 17.2	87.3 ± 10.0	<0.001

LPSPECT, lung perfusion single-photon emission computed tomography; BPA, balloon pulmonary angioplasty; WHO FC, world health organization functional classification; 6MWD, six-minute walk distance; BNP, brain natriuretic peptide; TRPG, tricuspid regurgitant pressure gradient; SvO_2_, mixed venous oxygen saturation; mPAP, mean pulmonary artery pressure; CI, cardiac index; PVR, pulmonary vascular resistance; FVL, functional %volume of lung

**Table 3:** Spearman’s correlation and multiple regression analyses for a dependent variable in mPAP

	Univariate		Multivariate	
	Correlation coefficients	*P* value	Standardized coefficients	*P* value
WHO FC	− 0.095	0.690		
6MWD	− 0.571	0.009	− 0.535	0.001
BNP	− 0.231	0.327		
TRPG	− 0.174	0.464		
PVR	0.315	0.176		
FLV-LPSPECT	− 0.728	< 0.001	− 0.565	< 0.001

mPAP, mean pulmonary artery pressure; WHO FC, world health organization functional classification; 6MWD, six-minute walk distance; BNP, brain natriuretic peptide; TRPG, tricuspid regurgitant pressure gradient; PVR, pulmonary vascular resistance; BPA, balloon pulmonary angioplasty; FVL-LPSPECT, functional %volume of lung-lung perfusion single-photon emission computed tomography

**Table 4:** Spearman’s correlation analysis between parameters and 6MWD

	Univariate	
	Correlation coefficients	*P* value
WHO FC	− 0.114	0.634
BNP	− 0.091	0.703
TRPG	0.207	0.381
PVR	− 0.150	0.527
mPAP	− 0.571	0.009
FLV-LPSPECT	0.074	0.757

6MWD, six-minute walk distance; WHO FC, world health organization functional classification; BNP, brain natriuretic peptide; TRPG, tricuspid regurgitant pressure gradient; PVR, pulmonary vascular resistance; mPAP, mean pulmonary artery pressure; BPA, balloon pulmonary angioplasty; FVL-LPSPECT, functional %volume of lung-lung perfusion single-photon emission computed tomography

**Table 5:** Spearman’s correlation analysis between parameters and PVR

	Univariate	
	Correlation coefficients	*P* value
WHO FC	− 0.397	0.083
6MWD	− 0.150	0.527
BNP	− 0.107	0.654
TRPG	0.105	0.661
mPAP	0.315	0.176
FLV-LPSPECT	− 0.361	0.118

PVR, pulmonary vascular resistance; WHO FC, world health organization functional classification; 6MWD, six-minute walk distance; BNP, brain natriuretic peptide; TRPG¸tricuspid regurgitant pressure gradient; BPA, balloon pulmonary angioplasty; FVL-LPSPECT, functional %volume of lung-lung perfusion single-photon emission computed tomography

### Case presentation

**Figure 3:**
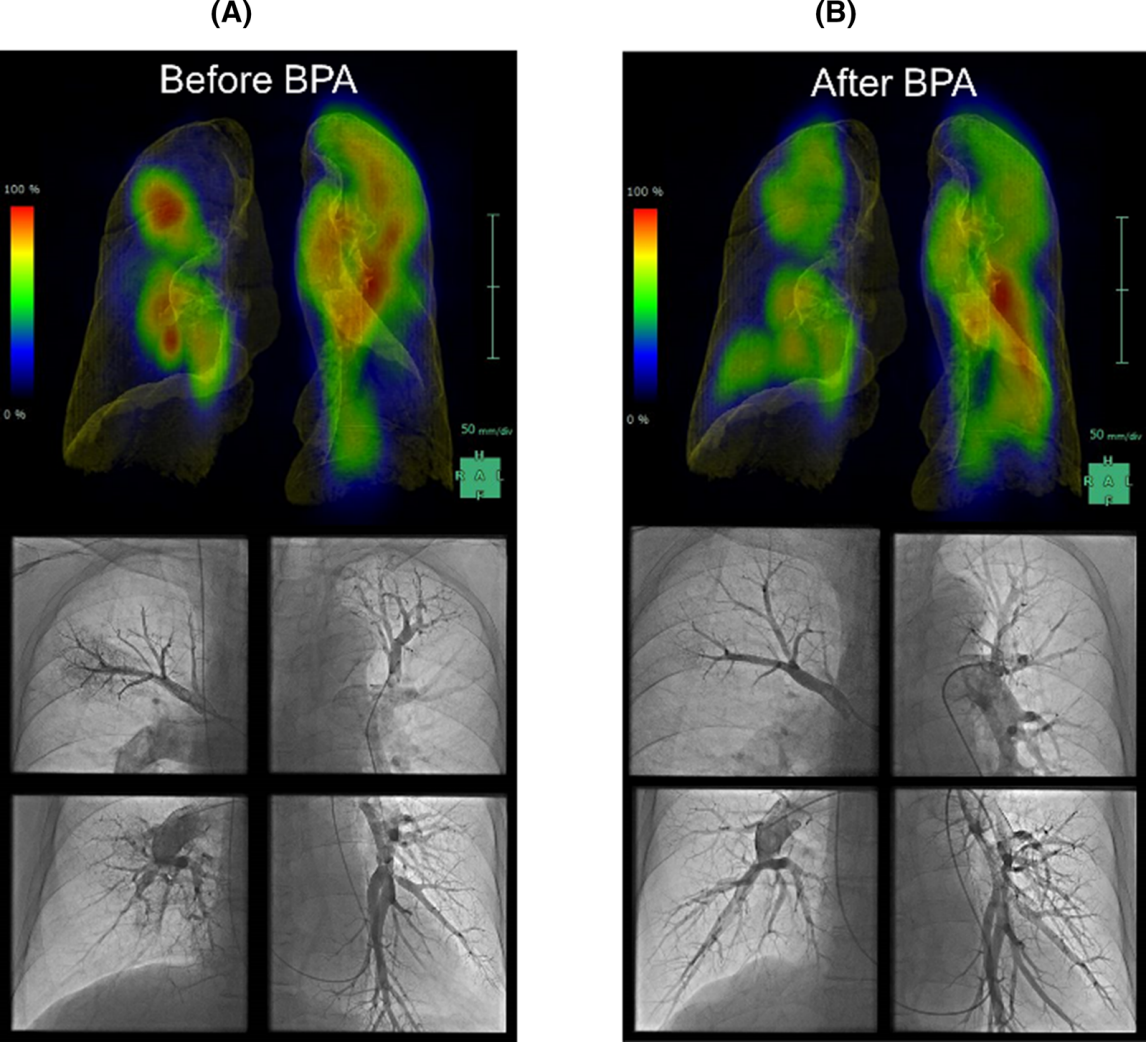
SPECT and CT fusion images and pulmonary artery angiographic images of a patient with CTEPH before and after BPA. SPECT, single-photon emission computed tomography; CT, computed tomography; CTEPH, chronic thromboembolic pulmonary hypertension; BPA, balloon pulmonary angioplasty

Figure 3 shows the SPECT and CT fusion images and pulmonary artery angiographic images of a 61-year-old man with CTEPH (WHO FC: 3, 6MWD: 360 m, PVR: 476.2 dyne sec cm^-5^, mPAP: 32 mmHg). He underwent lung perfusion SPECT and chest CT for imaging evaluation. Figure 3(a) upper image shows the lung perfusion SPECT and CT fusion image before BPA. There were perfusion defects in the upper and lower lobes of the right lung and the lower lobe of the left lung. The FVL-LPSPECT was 63.7%. Figure 3 lower image shows the pulmonary artery angiographic image before BPA. There were subtotal lesions in A1, A5, A9, and A10 and web lesions in A2, A3, A4, A6, A7, and A8 in the right pulmonary artery and web lesions in A1, A3, A4, A5, and A6 and ring-like lesions in A2, A8, A9, and A10 in the left pulmonary artery. During five sessions of BPA were performed, 56 branches were treated. Figure 3(b) lower image shows that the perfusion of the right ascending pulmonary arteries and both descending pulmonary arteries significantly improved after BPA. Figure 3(b) upper image shows that the perfusion defects in the upper and lower lobes of the right lung and lower lobe of the left lung improved after BPA. His WHO FC was 1, 6MWD was 435 m, PVR was 232.0 dyne sec cm^−5^, mPAP was 19 mmHg, and FVL-LPSPECT was 85.0% after BPA. In this case, all the clinical parameters showed improvement.

## Discussion

To our knowledge, this is the first study to objectively evaluate the effectiveness of BPA in patients with CTEPH using imaging modalities. The findings of the present study demonstrated that FVL-LPSPECT calculated using lung perfusion SPECT and CT could be a potential measurement of the effectiveness of BPA in patients with CTEPH.

### Evaluation of the severity of CTEPH

The parameters for measuring the effectiveness of BPA include mPAP, PVR, and 6MWD.^12^ mPAP has not only diagnostic but also prognostic value.^13,14^ A 3-year death rate of 90% was observed in patients with mPAP > 30 mmHg.^13^ The 2-year survival of patients with mPAP > 50 mmHg is less than 20%.^14^ Many studies have demonstrated that a decrease in PVR following the treatment of CTEPH leads to an improvement in prognosis.^15^ However, PVR does not always reflect irreversible pulmonary vascular disease. In left heart failure, the PVR might increase due to remodeling of the pulmonary vasculature and due to the effects of elevated left-sided filling pressures. This increased venous pressure also leads to higher vascular stiffness or lower vascular compliance. The lower compliance leads to enhanced pulmonary arterial wave reflections, which result in an increased systolic pulmonary artery pressure, and therefore mPAP.^16^ However, in this study, PVR was decreased after BPA, the parameter did not correlate with mPAP (Tables 2, 3, 5). PVR could not be accurately evaluated for pulmonary circulation due to the effects of blood flow and pressure. The 6MWD was reported as the primary endpoint to assess the clinical status and pulmonary hemodynamic parameters in most clinical trials. In some of these trials, improvement in 6MWD was accompanied by a parallel improvement in hemodynamic parameters,^17–20^ while other studies failed to demonstrate simultaneous changes in the 6MWD and hemodynamic status.^21,22^ Savarese et al. reported that changes in the 6MWD do not reflect the occurrence of an event and should only be considered indicative of the effects of therapies on the functional status.^23^ Therefore, mPAP is the most reliable marker for evaluating the efficiency of BPA and the prognosis of patients with CTEPH. In this study, as a result of multiple regression analysis for a dependent variable in mPAP, FVL-LPSPECT was the most significant parameter. FVL-LPSPECT can evaluate the pulmonary circulation without the influence of other pulmonary hemodynamic parameters and has a prognostic value in patients with CTEPH. Derlin et al. reported that quantitative analysis of threshold-based segmentation of perfused lung volumes using SPECT images provides a direct measure of the severity and correlates well with the established clinical parameters in patients with CTEPH.^24^ Maruoka et al. reported that a quantitative index of pulmonary perfusion scintigraphy using fractal analysis could predict therapeutic success after BPA in patients with CTEPH, and pulmonary perfusion volume using SPECT images is a valuable parameter for quantifying the therapeutic effect of BPA, visually and objectively.^25^ In their study, there was no significant difference in the perfusion volume calculated using SPECT between the low mPAP group and high mPAP group. Due to differences in physique, the perfusion volume at baseline was different between patients. In this study, FVL-LPSPECT was corrected using each CT volume for accurate representation of pulmonary circulation. Furthermore, Hosokawa et al. reported that lung perfusion SPECT on 3-dimensional CT angiography could delineate not only obstructive lesions but also hemodynamically significant web lesions that are often undetectable on angiography.^26^ Therefore, lung perfusion SPECT is a valuable imaging modality, which can act as a marker for deciding the treatment of pulmonary artery lesions and aid in predicting the efficacy of BPA using FVL-LPSPECT.

### Study limitations

This study has some limitations. First, the number of patients was relatively small, which limited the statistical reliability of the study. However, our results demonstrated that FVL-LPSPECT was significantly correlated with mPAP. Second, the cutoff level of the maximum radioactivity value in ^99m^Tc-MAA SPECT image analysis for calculating FVL-LPSPECT was based on visual observation in healthy patients. Further studies are necessary to confirm the adequate cutoff level. Third, the timing of image acquisition with each modality was different in the breathing phase; this might lead to lung volume differences between SPECT and CT. In this study, the images of SPECT and CT were taken in free-breath to reduce the volume difference. Fourth, the timing of enforcement of each modality was different. Future prospective studies with large populations are needed to confirm the correlation between FVL-LPSPECT and parameters of pulmonary circulation including mPAP, and the prognostic value of FVL-LPSPECT in patients with CTEPH.

## New Knowledge Gained

Lung perfusion SPECT is a valuable imaging modality that can be used during decision making related to the treatment of pulmonary artery lesions and for the measurement of the effectiveness of BPA.

## Conclusion

In this study, FVL-LPSPECT calculated using lung perfusion SPECT could potentially measure the effectiveness of BPA in patients with CTEPH.

## Supplementary Information

Below is the link to the electronic supplementary material.

Supplementary file1 (PPTX 317 kb)

Supplementary file1 (MP3 8268 kb)

## Abbreviations

CTEPH: Chronic thromboembolic pulmonary hypertension
BPA: Balloon pulmonary angioplasty
SPECT: Single-photon emission computed tomography
CT: Computed tomography
mPAP: Mean pulmonary arterial pressure
PVR: Pulmonary vascular resistance
^99m^Tc-MAA: Technetium-99m-macroaggregated albumin
FVL-LPSPECT: Functional %volume of lung calculated from the lung perfusion SPECT
6MWD: Six-minute walk distance
WHO FC: World health organization functional classification
