# Supplementary material for: Evaluation of balloon pulmonary angioplasty using lung perfusion SPECT in patients with chronic thromboembolic pulmonary hypertension
Source: J Nucl Cardiol. 2022 Apr 26;29(6):3392–400. doi: 10.1007/s12350-022-02971-0 (PMC9834092; doi:10.1007/s12350-022-02971-0)
Supplement: Supplementary file 1 — Supplementary file1 (PPTX 317 kb) [file 12350_2022_2971_MOESM1_ESM.pptx]

## Slide 1
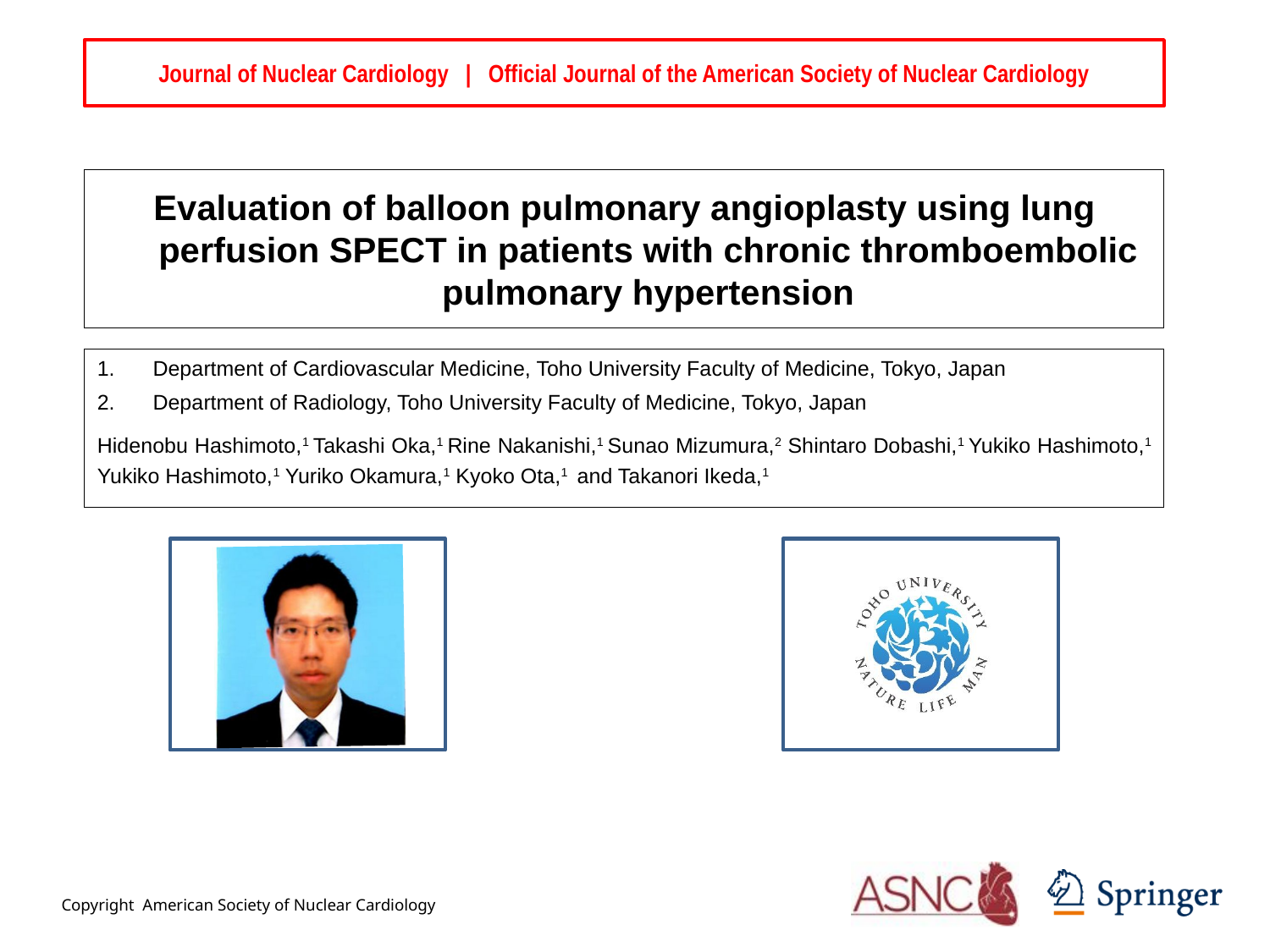

Journal of Nuclear Cardiology | Official Journal of the American Society of Nuclear Cardiology
# Evaluation of balloon pulmonary angioplasty using lung perfusion SPECT in patients with chronic thromboembolic pulmonary hypertension
Department of Cardiovascular Medicine, Toho University Faculty of Medicine, Tokyo, Japan
Department of Radiology, Toho University Faculty of Medicine, Tokyo, Japan
Hidenobu Hashimoto,1 Takashi Oka,1 Rine Nakanishi,1 Sunao Mizumura,2 Shintaro Dobashi,1 Yukiko Hashimoto,1 Yukiko Hashimoto,1 Yuriko Okamura,1 Kyoko Ota,1 and Takanori Ikeda,1
Head shot of author
required
Institution
Picture/Logo
Optional
Copyright American Society of Nuclear Cardiology

## Slide 2
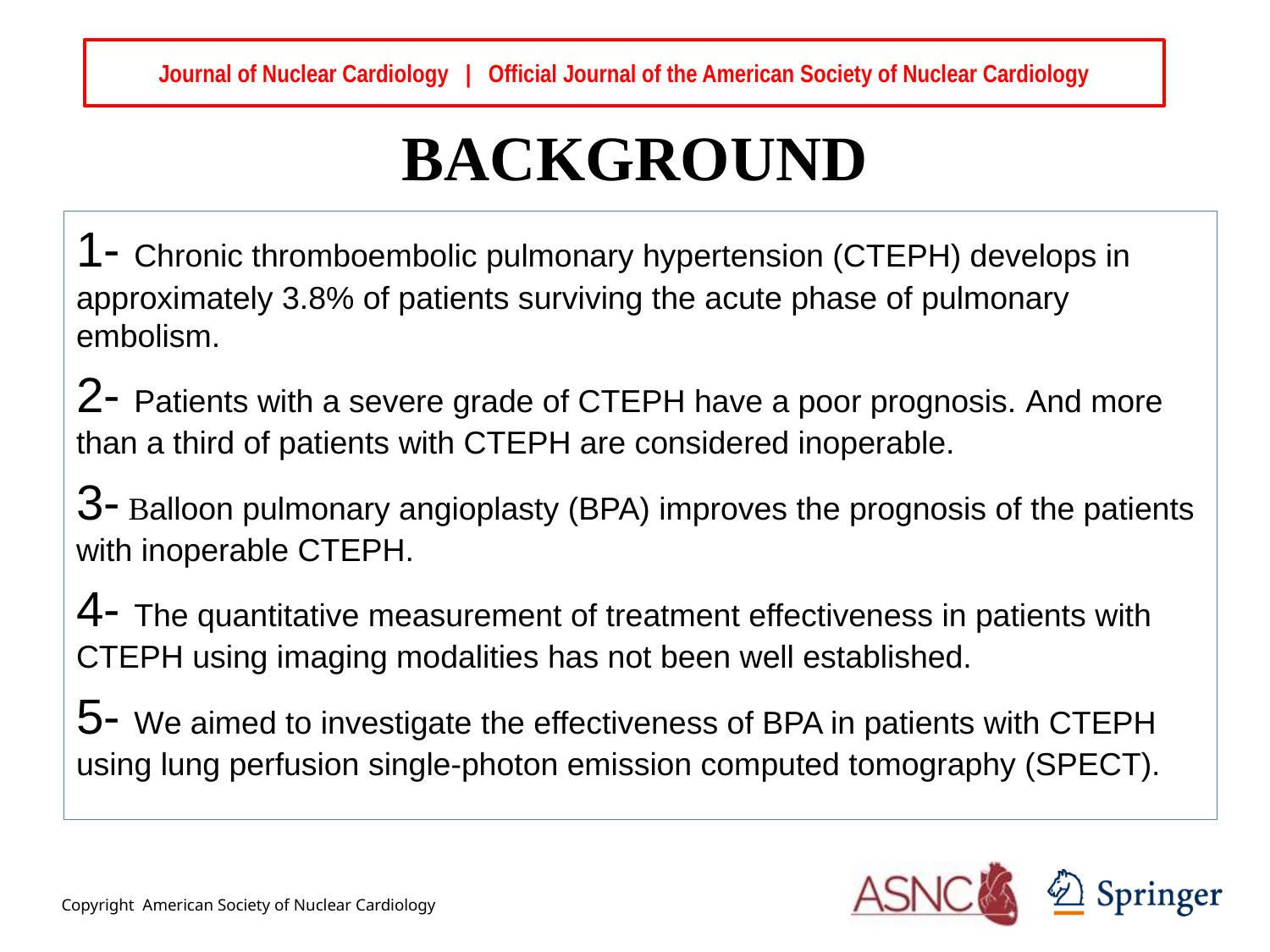

Journal of Nuclear Cardiology | Official Journal of the American Society of Nuclear Cardiology
# BACKGROUND
1- Chronic thromboembolic pulmonary hypertension (CTEPH) develops in approximately 3.8% of patients surviving the acute phase of pulmonary embolism.
2- Patients with a severe grade of CTEPH have a poor prognosis. And more than a third of patients with CTEPH are considered inoperable.
3- Balloon pulmonary angioplasty (BPA) improves the prognosis of the patients with inoperable CTEPH.
4- The quantitative measurement of treatment effectiveness in patients with CTEPH using imaging modalities has not been well established.
5- We aimed to investigate the effectiveness of BPA in patients with CTEPH using lung perfusion single-photon emission computed tomography (SPECT).
Copyright American Society of Nuclear Cardiology

## Slide 3
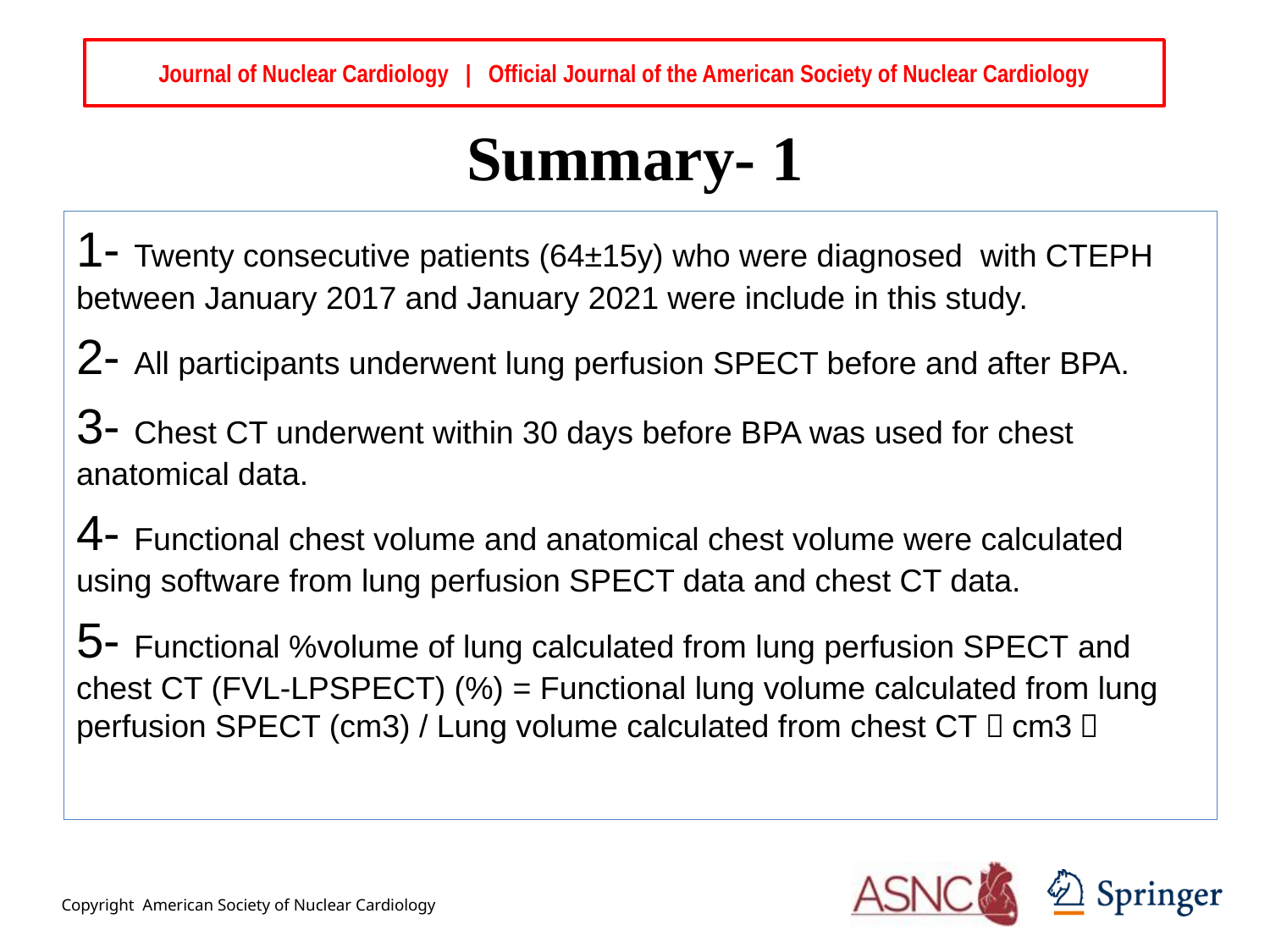

Journal of Nuclear Cardiology | Official Journal of the American Society of Nuclear Cardiology
# Summary- 1
1- Twenty consecutive patients (64±15y) who were diagnosed with CTEPH between January 2017 and January 2021 were include in this study.
2- All participants underwent lung perfusion SPECT before and after BPA.
3- Chest CT underwent within 30 days before BPA was used for chest anatomical data.
4- Functional chest volume and anatomical chest volume were calculated using software from lung perfusion SPECT data and chest CT data.
5- Functional %volume of lung calculated from lung perfusion SPECT and chest CT (FVL-LPSPECT) (%) = Functional lung volume calculated from lung perfusion SPECT (cm3) / Lung volume calculated from chest CT（cm3）
Copyright American Society of Nuclear Cardiology

## Slide 4
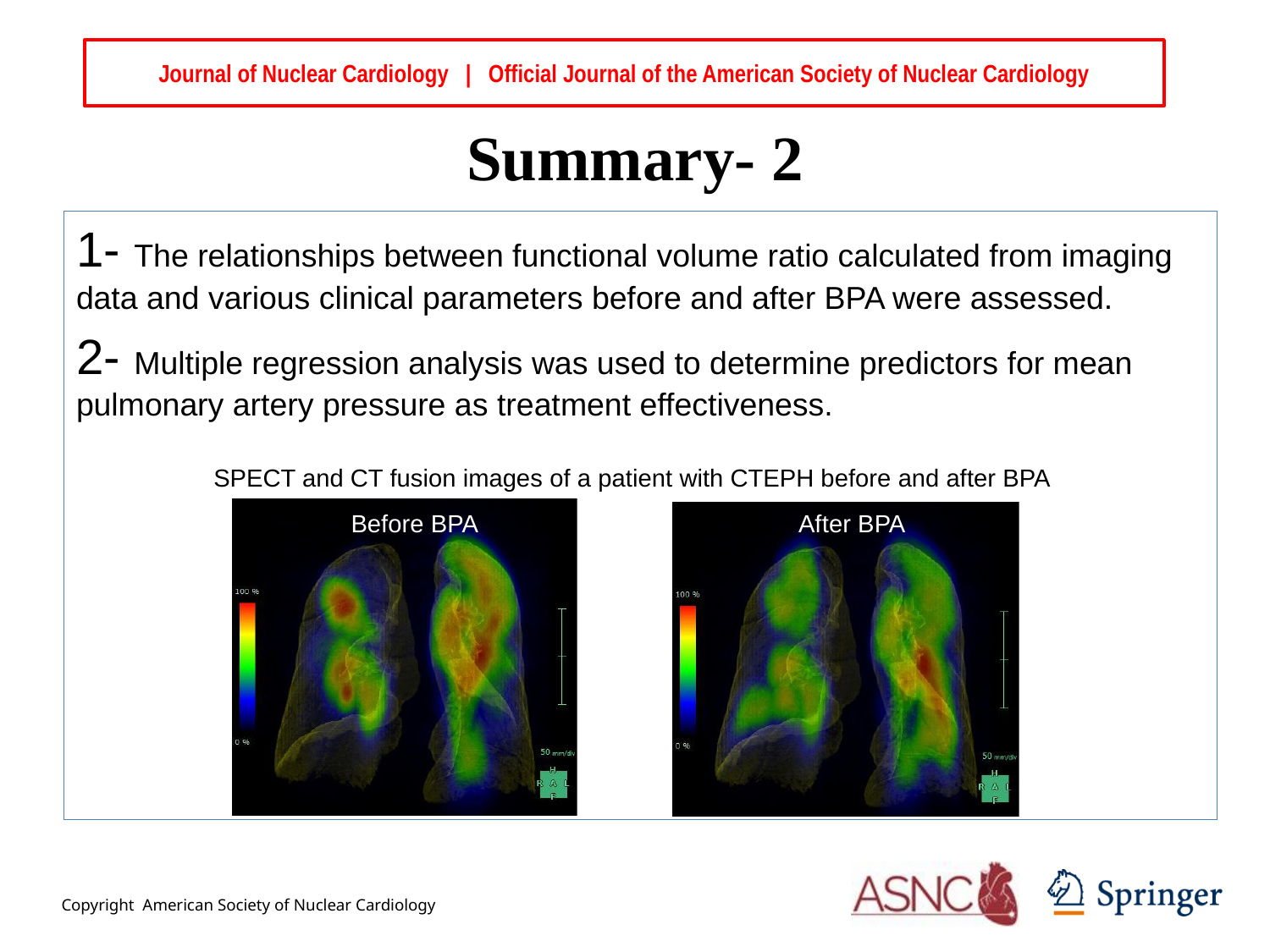

Journal of Nuclear Cardiology | Official Journal of the American Society of Nuclear Cardiology
# Summary- 2
1- The relationships between functional volume ratio calculated from imaging data and various clinical parameters before and after BPA were assessed.
2- Multiple regression analysis was used to determine predictors for mean pulmonary artery pressure as treatment effectiveness.
SPECT and CT fusion images of a patient with CTEPH before and after BPA
Before BPA
After BPA
Copyright American Society of Nuclear Cardiology

## Slide 5
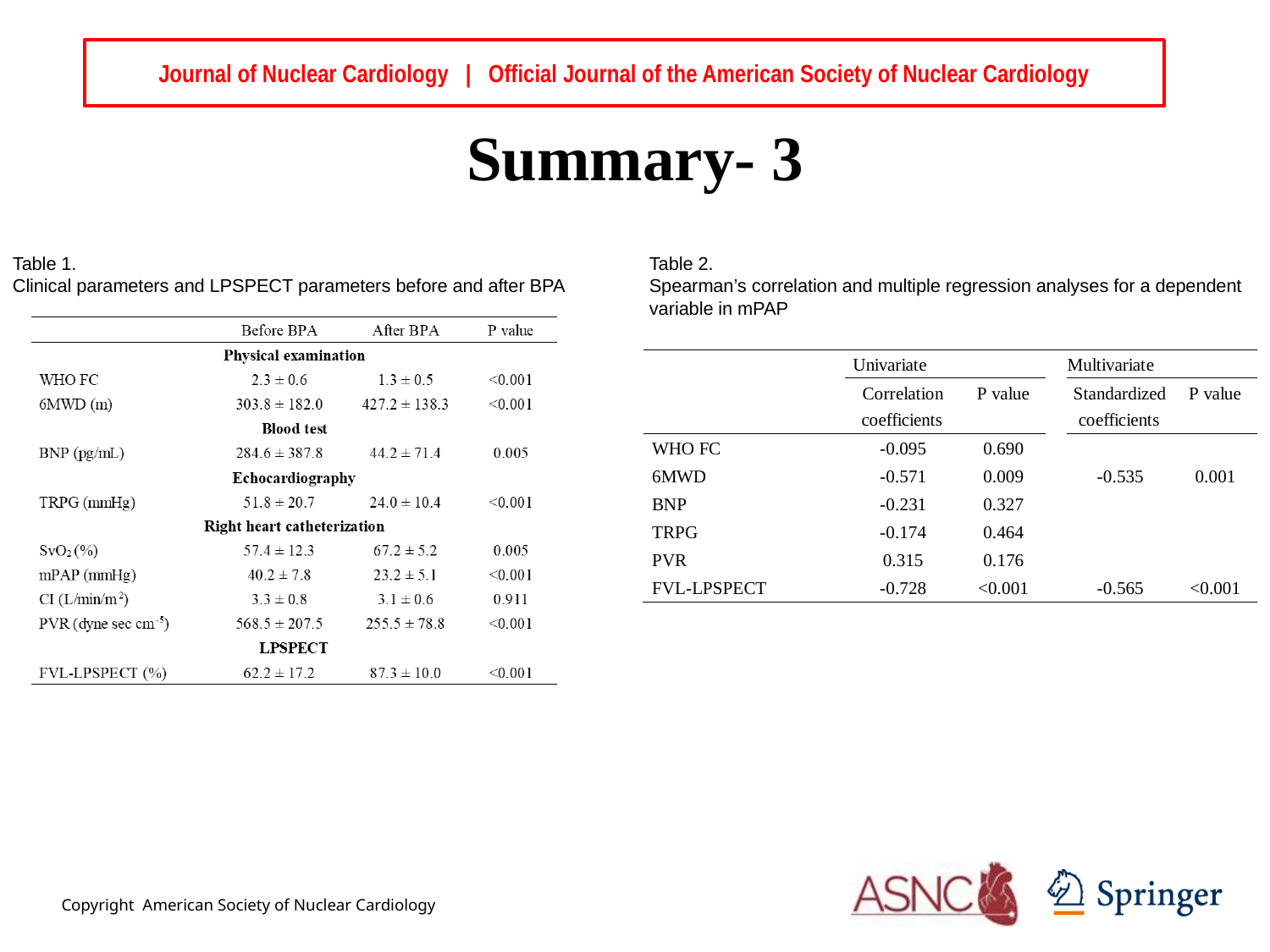

Journal of Nuclear Cardiology | Official Journal of the American Society of Nuclear Cardiology
# Summary- 3
Table 1.
Clinical parameters and LPSPECT parameters before and after BPA
Table 2.
Spearman’s correlation and multiple regression analyses for a dependent variable in mPAP
Copyright American Society of Nuclear Cardiology

## Slide 6
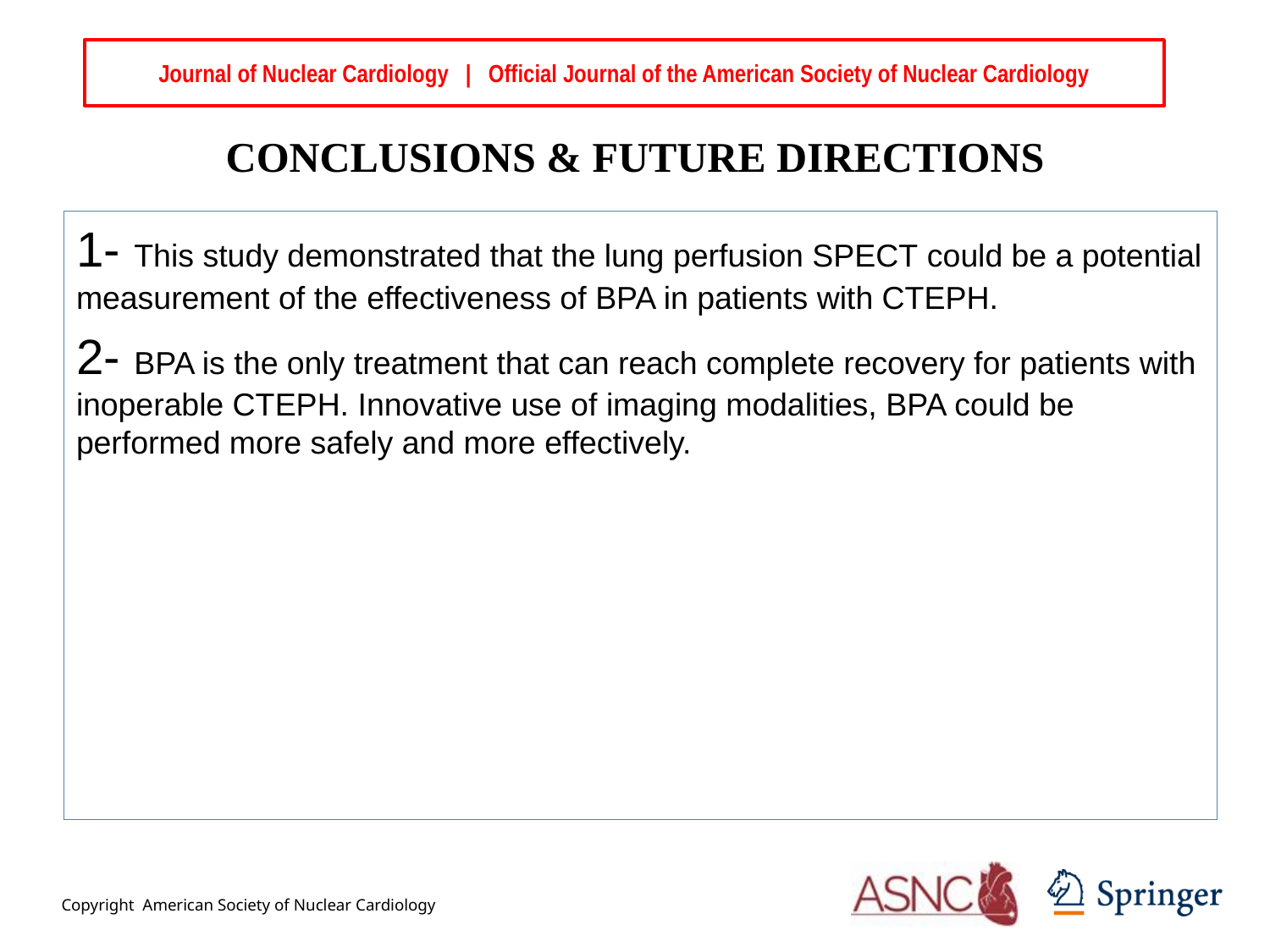

Journal of Nuclear Cardiology | Official Journal of the American Society of Nuclear Cardiology
# CONCLUSIONS & FUTURE DIRECTIONS
1- This study demonstrated that the lung perfusion SPECT could be a potential measurement of the effectiveness of BPA in patients with CTEPH.
2- BPA is the only treatment that can reach complete recovery for patients with inoperable CTEPH. Innovative use of imaging modalities, BPA could be performed more safely and more effectively.
Copyright American Society of Nuclear Cardiology
